# The Influence of Anteroposterior Head Inclination on the Perceived Consonance of the Smile Arc and Lower Lip Curvature on Photographs: A Cross-Sectional Study

**DOI:** 10.3390/jcm14051658

**Published:** 2025-02-28

**Authors:** Amir Reza Khadem, Matteo Togninalli, Gregory S. Antonarakis, Cristina Vento

**Affiliations:** 1 Division of Orthodontics, University Clinics of Dental Medicine, University of Geneva, 1211 Geneva, Switzerland; gregory.antonarakis@unige.ch (G.S.A.); cristina.vento@unige.ch (C.V.); 2 Visium SA, 1008 Prilly, Switzerland; matteo.togninalli@visium.ch

**Keywords:** smile arc, head position, smile consonance

## Abstract

**Objectives:** To determine the extent to which anteroposterior head inclination influences smile arc curvature assessment on frontal photographs. **Materials and Methods:** Sixty-three young adults participated in this study. Each had five standardized frontal-view photographs captured with posed smiles at five anteroposterior head inclinations (−20°, −10°, 0°, +10°, +20°) using a cervical range of motion device. Two curves were traced per photograph: one following the shape of the lower lip and the other the incisal edge of the maxillary anterior teeth from canine to canine (smile line). These curvatures were approximated by quadratic function and compared for concordance based on the maximum curvature of the obtained functions. A score was calculated, with 0 denoting a consonant smile (perfect concordance) and 2 a non-consonant smile. **Results:** Among the sixty-three participants, fifty-nine were included in the analysis after excluding those with insufficient tooth exposure in the photographs for the smile line assessment. The analysis revealed that the perceived smile line was more consonant (concordant with lower lip curvature) with a −20° head anteroposterior inclination (score: 0.146), and the least consonant with +20° anteroposterior inclination (score: 1.326), with statistically significant differences (*p* < 0.05). **Conclusions:** The smile arc curvature assessment on frontal photographs may be influenced by the anteroposterior inclination of the head on frontal photographs. However, due to the two-dimensional nature of this study, further investigations incorporating three-dimensional imaging are recommended.

## 1. Introduction

Smile aesthetics are influenced by various factors, including lip–teeth relationships [1], such as smile arc curvature, which is crucial for a balanced smile [2,3]. The importance of this smile arc curvature in smile aesthetics has been discussed for decades. Frush and Fisher [4] proposed that there should be a harmony between the curvature of the edges of the maxillary anterior teeth (smile arc) and that of the upper border of the lower lip, a concept reiterated by Tjan et al. [5], who stressed the importance of their parallelism. Sarver [6] defines the smile arc as the relationship between the curvature of the incisal edges of the maxillary incisors and canines to the curvature of the lower lip in a posed smile. He describes the “ideal” or “consonant” smile arc as one where the maxillary anterior tooth curvature parallels the lower lip when smiling, while a “non-consonant” or “flat” smile arc is when the maxillary anterior tooth curvature is flatter than that of the lower lip when smiling.

Smile arc evaluation is typically conducted using two-dimensional photographs of posed or spontaneous smiles, captured in the natural head position (NHP). Introduced by Broca in 1862 [7], the NHP defines the head position when standing with a horizontal visual axis, indicating a natural, relaxed posture.

Research suggests that even slight deviations from the NHP can distort frontal photographic images, potentially affecting perceptions of tooth color [8] and possibly facial or smile attractiveness. Studies using computer simulations or adjusting cast orientation to alter the occlusal plane have demonstrated that these changes impact perceived smile arc curvature and attractiveness ratings [9,10]. Recent studies have further explored the impact of head inclination on smile perception and attractiveness. For instance, Ren et al. [11] and Soranzo et al. [12] demonstrated that variations in camera angles and head positioning significantly influence aesthetic judgments, reinforcing the need for standardized protocols in smile assessments. Moreover, anteroposterior head inclination has been suggested to alter the perceived consonance of the smile arc, highlighting the importance of further standardization in photographic analysis.

A 2023 study further demonstrated that head positioning affects smile perception, with three-dimensional imaging providing more comprehensive diagnostic insights compared to traditional two-dimensional photographs [13]. Additionally, research indicates that head inclination influences incisor exposure, directly impacting the perceived curvature of the smile arc and its overall harmony within the facial profile [14].

Beyond static positional changes, facial expression dynamics, such as smile onset speed and head tilt, also play a role in perceived authenticity and attractiveness. Notably, slower smile onsets combined with specific head tilts have been found to enhance perceived genuineness [15].

Regarding the reproducibility of natural head position, changes in the pitch axis (sagittal plane) are the least reproducible three-dimensionally [16]. Camera positioning is also important, as it should be aligned parallel to the face [17]. Therefore, when evaluating the smile arc in two-dimensional photographs, whether for pre-treatment diagnosis or post-treatment evaluation, which is routinely performed, one must be attentive to the fact that the NHP may be important. An accurate and reproducible head posture might therefore be essential for capturing accurate extraoral photographs, as it minimizes distortion and provides a reliable basis for facial and smile analyses.

Multiple studies indicate that the smile arc significantly contributes to smile aesthetics, whereby those with a smile arc parallel to the lower lip curvature are perceived as having more attractive smiles [18,19,20,21,22] by orthodontists, dentists, and laypersons alike. Systematic review data affirm that the smile arc is a valid tool to assess the aesthetic appearance of a smile applied to both clinicians and laypersons [23]. The analysis of celebrities with highly rated smiles revealed similar smile arc characteristics [24], and self-reported smile satisfaction also correlates with smile arc consonance [25]. Optimal smile arcs thus exhibit parallel curvatures, while flattened arcs may indicate aging and compromised aesthetics [6,17,18]. Research emphasizes preserving the natural smile arc during orthodontic treatment to meet increasing aesthetic demands [6,17,18,26]. Given that head inclination and camera angle can influence smile arc perception [10,17], their potential impact on smile and facial attractiveness warrants further investigation.

The aim of the current study was to investigate the influence of the capture angle of posed smile frontal facial photographs, by altering the head position in the sagittal plane, on the evaluation of the consonance of the smile arc.

## 2. Materials and Methods

The present study, approved by the University Research Ethics Commission of Geneva (CUREG) under the reference number CUREG-MM-2022-11-152, was conducted from December 2022 to April 2023 at the University of Geneva, Switzerland. Participants provided written informed consent after being briefed on the procedures.

### 2.1. Sample and Methods

The sample consisted of sixty-three students and collaborators of the University clinics of dental medicine (Geneva, Switzerland). Age, gender, and orthodontic history were recorded. Inclusion criteria: Participants aged 18–40 years, in good general health, with visible maxillary anterior teeth. Exclusion criteria: Severe malocclusion, dental prostheses, significant facial trauma or surgery, deficient facial expressions or movements, craniofacial anomalies (e.g., cleft lip), or an inadequately visualized smile arc on photographs.

An a priori power analysis determined a sample size of fifty four was necessary for a moderate effect size (0.5) with a power of 0.8 and α = 0.05; thus, sixty-three participants were included to account for potential exclusions during the analysis if landmark location was not possible on the photographs.

### 2.2. Photography Protocol

**Figure 1:**
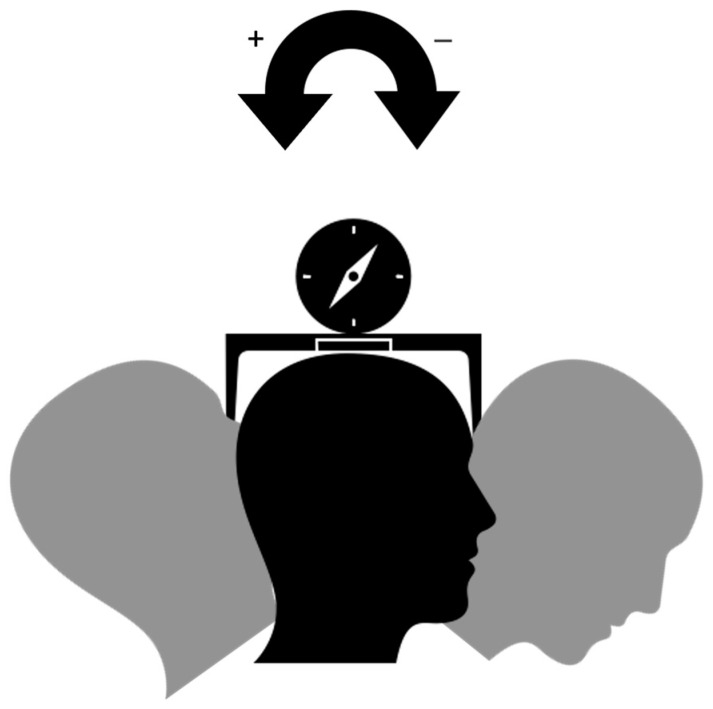
Changes in head inclination in the pitch axis with either negative or positive alterations, using the cervical range of motion device.

Five frontal facial photographs with a posed smile were taken for each participant, with pre-defined and quantifiable changes in the pitch axis (changes in the sagittal plane), namely at −20°, −10°, 0°, +10°, and +20° (Figure 1). Head orientation adjustments were achieved using a cervical range of motion (CROM) device [27], which ensures precise control over the pitch axis without altering the roll or yaw axes. This device, worn like glasses, includes two inclinometers (on the forehead and temporal area) to assess the pitch and roll, and a compass with shoulder magnets to monitor yaw positioning.

**Figure 2:**
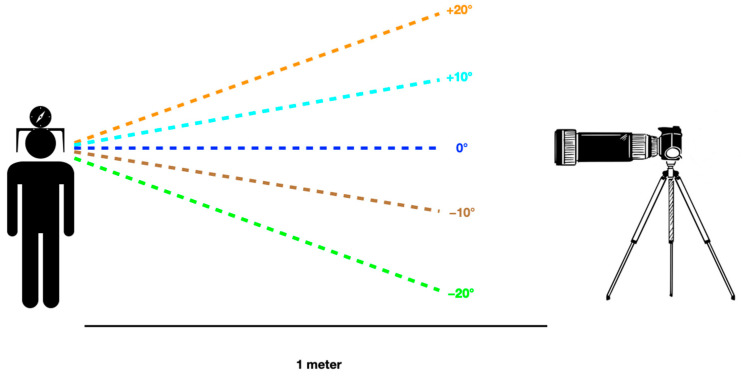
Setup for the photographic recording of the participants, at the five different head positions.

Photographs were taken using a standardized setup. A Canon EOS 90D camera with a 100 mm objective lens, connected via Bluetooth to a smartphone, allowed for real-time verification of each participant’s head position. The camera, mounted on a tripod and stabilized at 0° across the three axes, was placed one meter from the participant while taking the photographs (Figure 2).

### 2.3. Data Analysis

**Figure 3:**
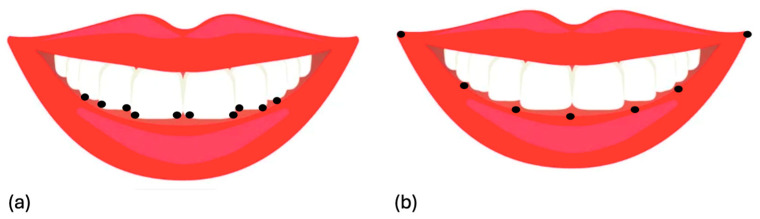
Reference points for defining curvature: (**a**) smile arc: mesial and distal edges of maxillary incisors and cuspids of both maxillary canines; (**b**) lower lip: right and left labial commissures, central midpoint, and two equidistant points between center and commissures.

Photographs were cropped to display only the lower third of each participant’s face, focusing on the smile arc and lower lip curvature. GNU Image Manipulation Program (GIMP 2.10; available from www.gimp.org accessed on 1 April 2023) software was used for the subsequent analysis. Reference points were directly located on the images representing the smile arc as well as the lower lip as follows:

- Smile arc: Ten reference points defined the smile arc curvature, including the mesial and distal edges of each maxillary incisor (two points per incisor) and the cuspids of the right and left maxillary canines (Figure 3a).
- Lower lip curvature: Seven points defined the lower lip curvature, including the right and left labial commissures, a central midpoint, and two equidistant points between the center and each commissure (Figure 3b).

The obtained points were then transformed into a consistent linear sequence utilizing Python scripts and libraries (numpy v1.23.4, pandas v1.5.1, svgpathtools v1.6.1) by an independent researcher who was not involved in the taking of the photographs. The scripts processed the traced points to generate two distinct quadratic functions, mathematically approximating the lower lip and the smile arc, respectively. The comparative analysis of these curves hinged on assessing the maximum curvature points derived from the quadratic functions. The maximum curvature, *κ*, of a quadratic function, *f*(*x*) = *ax*^2^ + *bx* + *c*, is given by its second derivative: *κ* = 2*a*. The degree of concordance between the two curves was calculated and expressed as a continuous value, ranging from 0 to 2, according to the following formula:

$$Consonance\;Score=\frac{\left|\kappa_{smile}-\kappa_{lip}\right|}{max(\left|\kappa_{smile}\right|,\left|\kappa_{lip}\right|)}$$

**Figure 4:**
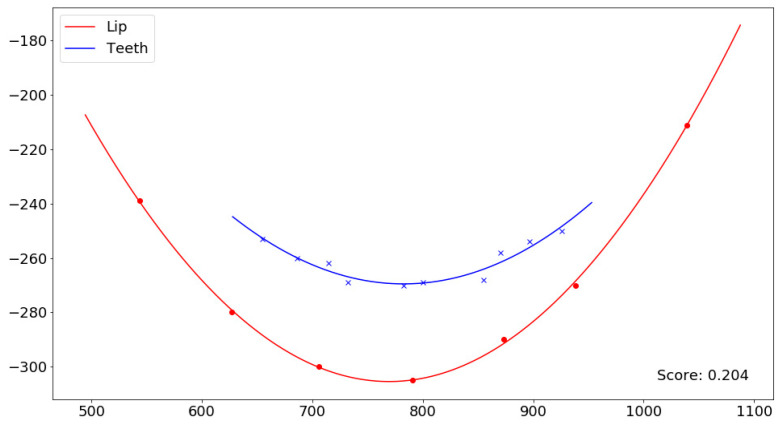
The red curve represents the lower lip curve, and the blue curve represents the smile arc. In the bottom right corner, the consonance score between these two curves is shown.

This score provides a quantifiable measure of smile consonance: values approaching 0 indicate a high concordance (with 0 representing a perfect concordance), representing a scenario where the smile line closely parallels the curvature of the lower lip, thus denoting a consonant smile. Conversely, values nearing 2 signify a lower degree of concordance (with 2 representing an inverse concordance), indicating a divergence between the smile line and the lower lip curve and thus a non-consonant smile. All values above 1 indicate reverse curves for the lower lip and smile line. In Figure 4, an example is shown where the red curve represents the lower lip curve and the blue curve represents the smile arc, along with their consonance score.

### 2.4. Statistical Analysis

To evaluate the reliability of the method, repeated analyses were conducted on five randomly selected participants by two operators performing the analyses twice independently. Pearson’s and Spearman’s correlation coefficients indicated high inter- and intra-rater reliability values (r > 0.980).

Main analysis: Consonance scores between the smile arc and lower lip curvature were calculated for each photograph at the various head inclinations. To examine the overall effect of head inclination on the consonance scores and determine whether the differences in the mean consonance scores between the head inclination angles were statistically significant, a one-way analysis of variance (ANOVA) was performed.

Following the ANOVA, post hoc tests were conducted to identify specific pairwise differences between head inclination angles. Both Tukey and Bonferroni post hoc tests were applied to adjust the significance threshold, reducing the risk of type I errors.

Linear regression: The relationship between head inclination angles and consonance scores was explored using a linear regression analysis, providing further insight into how changes in head position affected smile consonance.

Gender-specific analysis: To investigate potential gender differences, normality tests (Shapiro–Wilk) were performed, followed by *t*-tests or Mann–Whitney U tests based on the data distribution. Separate linear regression analyses were also conducted for males and females to explore whether the relationship between head inclination and consonance scores varied by gender.

All statistical analyses and graphical representations were carried out and generated using Python 3.0 (Python Software Foundation, Wilmington, DE, USA).

## 3. Results

### 3.1. Sample

Out of sixty-three initial participants, four were excluded due to the inadequate visibility of the smile arc, leaving fifty-nine participants (thirty-nine females and twenty males). Seventy-three percent (*n* = 43) had prior orthodontic treatment.

### 3.2. Data Analysis for the Whole Dataset

**Figure 5:**
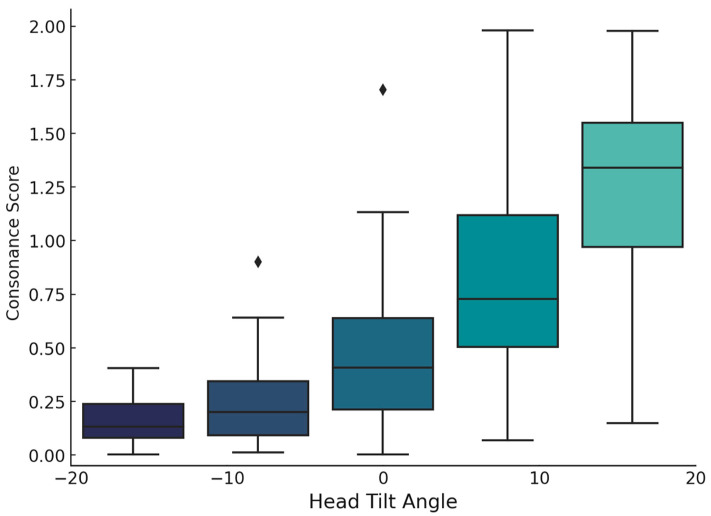
Box plots showing the distribution of consonance scores obtained for each head inclination angle for the whole sample.

Smile to lower lip consonance scores varied with head inclination angles from −20° to +20° (Figure 5). Higher head tilts were associated with increased mean consonance scores, with greater score variability observed at positive inclinations. The ANOVA results confirm a significant effect of head inclination on the consonance scores, F(4, 290) = 106.96, *p* < 0.001, indicating statistically significant differences in the consonance scores among the different head inclination groups.

Post hoc analyses using Tukey’s test showed that nearly all pairwise comparisons had *p*-values < 0.001, indicating statistically significant differences between most inclination groups. However, the comparisons between −20° vs. −10° (*p* = 0.5736) and −10° vs. 0° (*p* = 0.0043) did not reach statistical significance after adjustments for multiple comparisons. These results suggest that changes in head inclination, particularly from negative to positive angles, are associated with notable differences in the consonance scores. The analysis further confirms that smile consonance tends to decrease as the head inclination angle increases, highlighting specific degrees where these changes become statistically significant.

**Figure 6:**
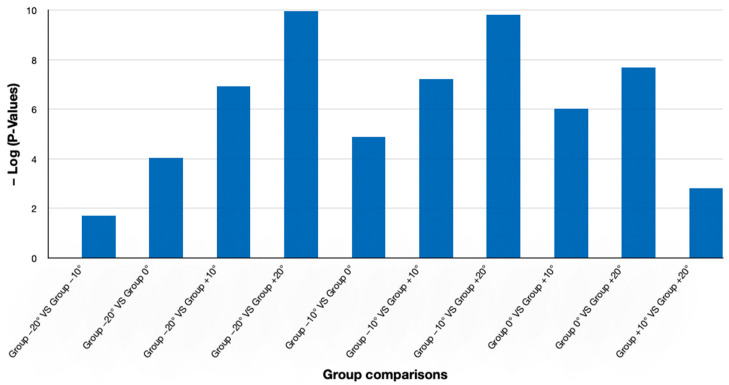
Bar chart of group comparison *p*-values, shown with a −log(*p*-value) transformation for clearer visualization due to extremely low values.

Further analysis using the Bonferroni post hoc test supported these findings, revealing significant differences between all comparisons (Figure 6). Interestingly, the comparison between −20° and −10°, which was not found to be significant in the Tukey test, showed a corrected *p*-value of 0.0194 in the Bonferroni test, indicating a significant difference. Additionally, other comparisons (e.g., −20° vs. 0°, −10° vs. +20°, etc.) were also found to be significantly different, with corrected *p*-values approaching zero. Overall, these findings confirm that most changes in head inclination lead to significant variations in the smile consonance scores, as evidenced by both post hoc approaches.

**Figure 7:**
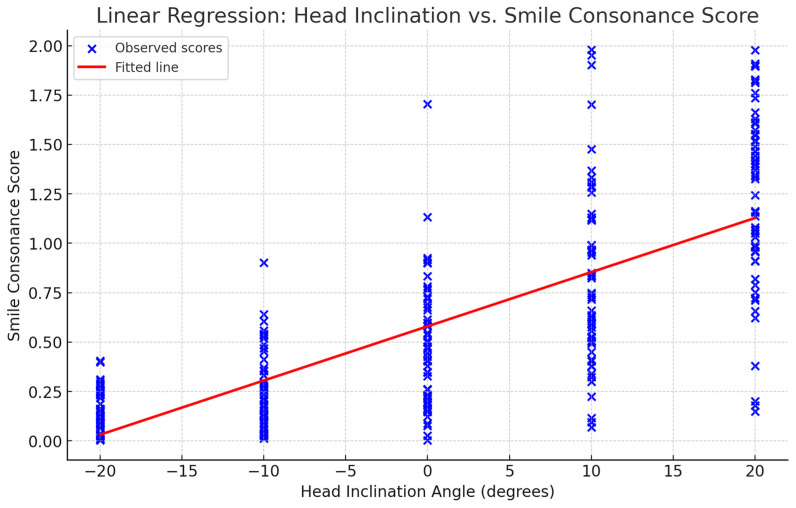
Linear regression curve illustrating the relationship between the head inclination angle and smile consonance score for the whole sample. Blue dots represent observed scores at each angle, while the red line indicates the trend: an increased head inclination correlates with a decreased smile consonance.

Linear regression analysis revealed a statistically significant positive association between head inclination and consonance scores (R^2^ = 0.562; β = 0.0274, *p* < 0.001), with each degree increase in inclination reducing consonance by an average score increment of 0.0274 (Figure 7).

### 3.3. Gender-Specific Data Analysis

**Figure 8:**
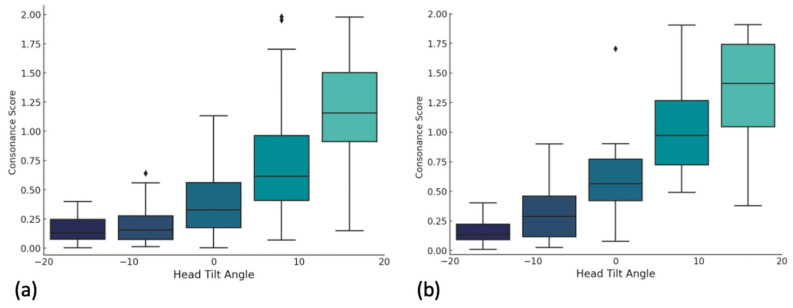
Box plots showing the distribution of consonance scores obtained for each head inclination angle for female (**a**) and male (**b**) participants separately.

Gender comparisons showed no significant differences at −20°, −10°, and 20° inclinations (Figure 8). However, males had significantly higher consonance scores at 0° (*p* = 0.010) and 10° (*p* = 0.006), suggesting a greater divergence between the smile arc and lower lip curvature at these angles compared to females.

**Figure 9:**
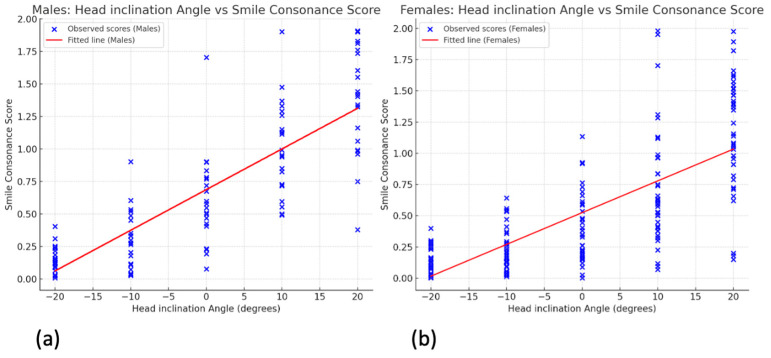
Linear regression curves illustrating the relationship between the head inclination angle and smile consonance score for (**a**) the male participants and (**b**) the female participants. Blue dots represent observed scores at each angle, while the red line indicates the trend: an increased head inclination correlates with a decreased smile consonance.

Separate regression analyses (Figure 9) found that, for males, each degree of head inclination correlated with a 0.0313 increase in the consonance scores (β1 = 0.0313, *p* < 0.001) with an intercept of 0.6878 (R^2^ = 0.664). For females, the increase per degree was 0.0254 (β1 = 0.0254, *p* < 0.001) with an intercept of 0.5242 (R^2^ = 0.529).

## 4. Discussion

The present study investigated the effect of anteroposterior head inclination on the perceived consonance between the smile arc and lower lip curvature in frontal photographs. The results indicate that a −20° head tilt yields the most consonant smile, while positive inclinations (+10° and +20°) show a significantly reduced consonance. These findings underscore the importance of head position in smile assessments, particularly in orthodontic record-taking and aesthetic evaluations.

These findings align with prior research demonstrating the influence of head inclination on the perception of facial aesthetics. Studies by Ren H. et al. [11] and Soranzo et al. [12] have shown that variations in capture angles affect perceptions of facial attractiveness, emphasizing the relevance of head positioning in photographic analyses.

The parameters of normality for teeth display in smile aesthetics were defined based on prior studies [5,6]. These studies describe an “ideal” smile arc as one where the curvature of the maxillary anterior teeth closely parallels the curvature of the lower lip during a posed smile. Deviations from this alignment are considered less aesthetically pleasing and were quantified in our study using a consonance scoring system. This approach provides an objective framework for evaluating smile aesthetics, minimizing subjectivity in the analysis.

Interestingly, the observed stronger influence of head inclination in males, as indicated by more consistent model fits, suggests potential gender-based differences in aesthetic perception. Future studies could explore whether these differences stem from cultural or perceptual biases, offering valuable insights into personalized aesthetic evaluations.

Several limitations of this study should be acknowledged. First, the sample comprised predominantly young adults, with a higher proportion of females. A more homogeneous sample, particularly including individuals with optimal smile lines and diverse demographic characteristics, would enhance the reliability and generalizability of the findings. Future research should aim for greater sample uniformity by focusing on participants with smile lines consonant with lower lip curvature to be able to better isolate the effects of head inclination.

Additionally, the reliance on two-dimensional photographs presents inherent limitations. The planar reduction of facial features may introduce bias, as static images fail to capture the dynamic nature of smiles and subtle facial expressions during a natural smile. This limitation can lead to an incomplete interpretation of smile aesthetics, as the precise moment when the photograph is taken during the smiling process may affect the perceived consonance between the smile arc and lower lip curvature. Incorporating three-dimensional imaging or video recordings could provide more accurate assessments of smile aesthetics, as these modalities capture head positioning and facial dynamics with greater precision. Such advanced imaging techniques would allow for a more comprehensive analysis of smile sequences from start to finish, potentially offering insights into how smile aesthetics change throughout the expression. Furthermore, three-dimensional or video analysis could help overcome the challenges in interpreting the results based on static images, which may affect the clinical application of the findings, particularly in planning orthodontic or aesthetic treatments. Future studies employing these technologies could enhance our understanding of smile aesthetics across different head positions and provide a more realistic representation of smiles in real-world conditions.

Another limitation is the inclusion of participants with prior orthodontic treatment, which may have introduced variability in smile aesthetics. While efforts were made to control for this variability, a future study with orthodontically naïve participants or stratification based on orthodontic history could provide clearer insights. Our sample included 69% (*n* = 41) participants with prior orthodontic treatment. While we did not specifically analyze differences between treated and non-treated individuals, previous research by Maganzini et al. [26] has shown that orthodontic treatment can improve smile aesthetics. Future studies could stratify participants based on orthodontic history to explore how treatment might interact with head inclination to affect smile consonance.

The subjective scoring of consonance may also introduce bias, influenced by cultural and individual differences [28]. To minimize this bias, future studies could use a more diverse panel of evaluators or implement a calibration method for evaluators to improve consistency. In the present study, however, an objective evaluation method was used in order to overcome the subjectivity of this assessment.

Despite its limitations, this study provides valuable insights into smile aesthetics and reinforces the importance of standardizing clinical photography. Our findings offer quantitative evidence demonstrating a systematic impact of head inclination on smile consonance, strengthening the scientific framework for evaluating head positioning in aesthetic assessments.

From a methodological perspective, this study enhances precision and reproducibility through automated mathematical modeling, reducing the subjectivity inherent in manual tracings. By analyzing a structured range of inclinations, it moves beyond simply highlighting the limitations of two-dimensional imaging, offering a clearer understanding of how head position alters smile perception.

The findings have key clinical implications for orthodontic practice and aesthetic dentistry, highlighting the importance of standardizing head position in extraoral photography for consistency and accuracy in assessments. Since head inclination markedly affects perceived smile consonance and aesthetics, practitioners should adopt consistent head positioning during photographic documentation to achieve reliable results.

However, achieving standardized head positioning in clinical settings can be challenging due to variability in patient comfort and photographic equipment settings. Developing guidelines or employing tools such as the cervical range of motion device used in this study could aid in achieving more consistent head positioning.

Incorporating three-dimensional imaging into routine practice could further enhance the precision of smile assessments. This technology would enable practitioners to offer patients a more realistic visualization of treatment outcomes, ultimately improving patient satisfaction by optimizing aesthetic presentations.

Our study serves as a foundation for future studies incorporating three-dimensional imaging and video-based analysis to better capture the dynamic nature of smiles. By addressing the limitations of static photography, our findings pave the way for more refined diagnostic and treatment planning strategies in orthodontics and aesthetic dentistry.

## 5. Conclusions

This study highlights that anteroposterior head inclination significantly affects perceived smile consonance in two-dimensional extraoral frontal photographs. A slightly negative head tilt enhances consonance, while a positive tilt reduces it. These findings underscore the importance of standardized head positioning in photography for consistent and accurate orthodontic assessments.

Future research incorporating three-dimensional or videographic imaging into daily orthodontic practice could address the limitations of two-dimensional photos, enhancing diagnostic quality and providing patients a more realistic view of aesthetic outcomes, thereby improving satisfaction.

## Data Availability

The data that support the findings of this study will be made available from the corresponding author upon reasonable request.
